# Thalamocortical input onto layer 5 pyramidal neurons measured using quantitative large-scale array tomography

**DOI:** 10.3389/fncir.2013.00177

**Published:** 2013-11-12

**Authors:** Jong-Cheol Rah, Erhan Bas, Jennifer Colonell, Yuriy Mishchenko, Bill Karsh, Richard D. Fetter, Eugene W. Myers, Dmitri B. Chklovskii, Karel Svoboda, Timothy D. Harris, John T. R. Isaac

**Affiliations:** ^1^Howard Hughes Medical Institute, Janelia Farm Research CampusAshburn, VA, USA; ^2^Developmental Synaptic Plasticity Section, National Institute of Neurological Disorders and Stroke, National Institutes of HealthBethesda, MD, USA; ^3^Department of Engineering, Toros UniversityMersin, Turkey

**Keywords:** array tomography, electron microscopy, thalamocortical synapse, dendritic integration, synapse distribution, barrel cortex, neural circuits

## Abstract

The subcellular locations of synapses on pyramidal neurons strongly influences dendritic integration and synaptic plasticity. Despite this, there is little quantitative data on spatial distributions of specific types of synaptic input. Here we use array tomography (AT), a high-resolution optical microscopy method, to examine thalamocortical (TC) input onto layer 5 pyramidal neurons. We first verified the ability of AT to identify synapses using parallel electron microscopic analysis of TC synapses in layer 4. We then use large-scale array tomography (LSAT) to measure TC synapse distribution on L5 pyramidal neurons in a 1.00 × 0.83 × 0.21 *mm*^3^ volume of mouse somatosensory cortex. We found that TC synapses primarily target basal dendrites in layer 5, but also make a considerable input to proximal apical dendrites in L4, consistent with previous work. Our analysis further suggests that TC inputs are biased toward certain branches and, within branches, synapses show significant clustering with an excess of TC synapse nearest neighbors within 5–15 μm compared to a random distribution. Thus, we show that AT is a sensitive and quantitative method to map specific types of synaptic input on the dendrites of entire neurons. We anticipate that this technique will be of wide utility for mapping functionally-relevant anatomical connectivity in neural circuits.

## Introduction

Cortical pyramidal neurons receive input via thousands of synapses distributed over the entire dendritic tree. The functional efficacy of synapses is influenced by their electrotonic distance from the soma, the presence of local active conductances, biochemical compartmentalization and proximity to co-active synapses at the μm scale (Polsky et al., 2004; London and Hausser, 2005; Yasuda et al., 2006; Harvey and Svoboda, 2007; Harvey et al., 2008). The subcellular location of synapses is therefore a critical factor in shaping their functional impact. On thalamo-recipient dendrites in primary sensory cortex, thalamocortical input accounts for only a small fraction of the total synapses, yet, functionally, the input is strong (Benshalom and White, 1986; Peters and Payne, 1993; Ahmed et al., 1994; Douglas et al., 1995; Alonso et al., 1996; Stratford et al., 1996; Gil et al., 1999; Beierlein et al., 2002; Bruno and Sakmann, 2006). The explanation for this anatomical-functional disparity is unclear. One prominent yet largely untested hypothesis (Larkum and Nevian, 2008) is that the dendritic location and clustering of the input could be an important factor in determining its strength.

The current lack of knowledge about the subcellular distribution of synapses is largely due to a lack of appropriate techniques. Calcium imaging can be used to detect active synapses; however, this approach lacks single synapse resolution and large-scale capacity (Petreanu et al., 2009; Richardson et al., 2009; Bagnall et al., 2011). Optogenetic approaches lack fine-scale resolution (Petreanu et al., 2009; see, however, Little and Carter, 2012). Another approach uses overexpressed GFP-fusions of interacting pre- and post-synaptic proteins such that fluorescence occurs only when they are in very close proximity (“GRASP”) (Feinberg et al., 2008; Kim et al., 2012). Although promising, this approach requires overexpression of synaptic proteins that may affect synaptic and network function (Scheiffele et al., 2000; Graf et al., 2004). Furthermore, GRASP relies on molecular targeting to synapses and thus may have to be redeveloped anew for different types of neurons.

The ideal technique requires the necessary sensitivity to reliably detect individual synapses and to resolve individual synapses. The method should be applicable to a sufficiently large volume of tissue to allow reconstruction of entire dendritic trees and needs to be combined with labeling of specific input. Synapses are easily resolved using electron microscopy (EM); however, this method is rarely used for reconstruction of very large volumes (Bock et al., 2011; Briggman et al., 2011; Takemura et al., 2013) and is not easily compatible with labeling methods while preserving ultrastructure. While conventional fluorescence light microscopy allows large-scale imaging and is compatible with labeling of specific synaptic inputs, its *Z*-axis (depth) resolution is insufficient for resolving synapses in tissue (Mishchenko, 2010).

We reasoned that array tomography (AT) has many of these critical attributes. AT is a high-resolution, wide-field fluorescence imaging technique based on repeated imaging of arrays of ultrathin serial sections, followed by computational reconstruction into a three-dimensional volume (Micheva and Smith, 2007; Micheva et al., 2010). The use of ultrathin sections enables isotropic resolution and reliable and repeatable immunostaining of synaptic proteins. Moreover, this technique can be combined with the use of molecular-genetic approaches to express fluorescent reporters in specified neuronal populations enabling labeling of synaptic inputs of specific origin. Finally, sufficiently large volumes can be imaged, potentially encompassing entire dendritic trees. It has not yet been determined if AT can reliably resolve individual synapses.

Here we characterize large-scale AT (LSAT) for mapping specific types of inputs within the dendritic arbors of individual neurons. We focus on TC input onto the dendritic trees of layer (L) 5 pyramidal neurons. We compare AT and EM 3D stacks to quantify the accuracy and reliability of AT synapse detection. We then perform LSAT (1.00 × 0.83 × 0.21 mm^3^ volume) on a block of mouse somatosensory cortex and locate TC synapses on the dendritic trees of a number of L5 pyramidal neurons. This data set suggests that TC input exhibits clustering and dendritic branch preference, and demonstrates the power and utility of this approach.

## Results

**Figure 1:**
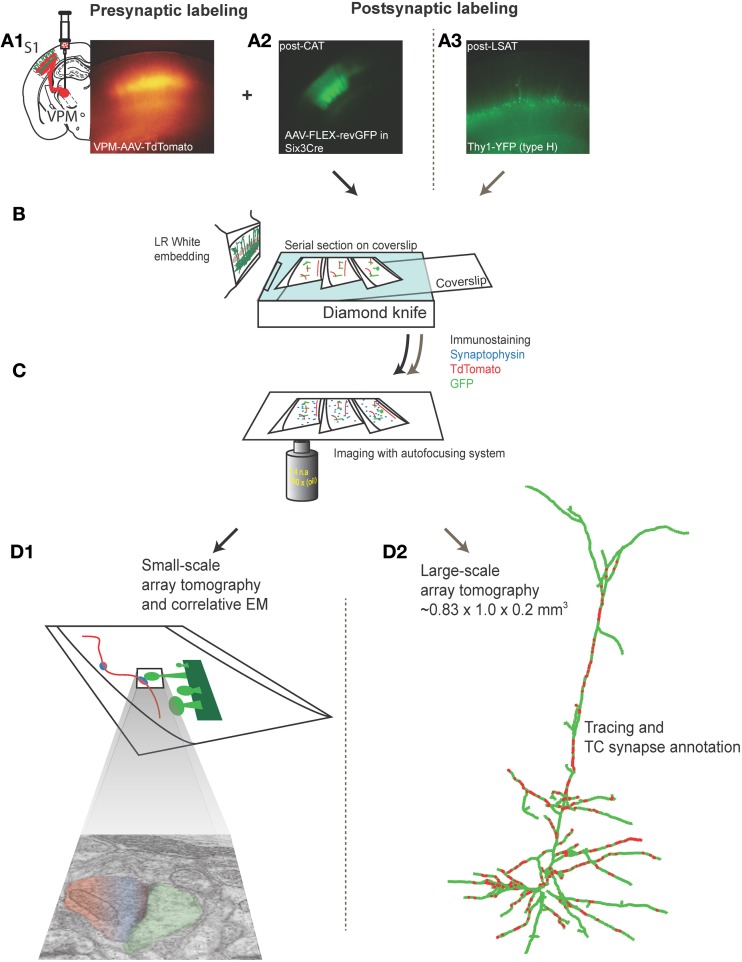
**Labeling and imaging thalamocortical synapses on L4 or L5 barrel cortex neurons using array tomography and electron microscopy. (A1)** Low power fluorescence photomicrograph of tdTomato-expressing TC axons and terminals in a fixed section of barrel cortex prepared from an 5–6 month old mouse in which AAV-tdTomato was stereotaxically injected in VPm 20–30 days earlier (inset left shows schematic of VPm injection). **(A2)** Low power fluorescence photomicrograph of GFP fluorescence in neurons in L4 barrel cortex in a fixed section from adult six3-CRE mouse in which AAV-FLEX-revGFP virus was injected into L4, 20-30 days earlier. **(A3)** Low power fluorescence photomicrograph of GFP fluorescence in L5 pyramidal neurons in fixed section from a 5–6 month old Thy1-YFP_h_ mouse. **(B)** Schematic of LR white embedding and ultrathin serial sectioning procedure. **(C)** Schematic of immunostaining and light imaging of serial sections. **(D1)** Schematic of light and electron microscopic imaging on the same sections to assess accuracy of synapse detection and annotation with array tomography (performed on VPm-L4 only). **(D2)** Reconstructed L5 pyramidal neuron and annotated TC synapses from large-scale array tomography experiment (VPm-L5 only).

To study the subcellular distribution of TC synapses onto L5 pyramidal neurons in primary somatosensory barrel cortex with LSAT, we labeled pre- and post-synaptic groups of neurons with different fluorescent proteins (Figure 1A). Post-synaptic neurons were labeled in Thy1-YFP (type H) mice in which cortical L5 neurons sparsely express YFP (Feng et al., 2000). We labeled neurons in the ventral posteriomedial nucleus of thalamus (VPm), which project to barrel cortex, with adeno-associated virus (AAV) expressing tdTomato. Adult mice were stereotaxically injected with AAV. After ~4 weeks of expression the brain was fixed by transcardial perfusion. The primary somatosensory cortex was embedded in LR White resin. Serial ultrathin sections (200 nm) were made from the embedded block covering a volume of ~0.2 mm^3^. Each section was stained with an anti-synaptophysin antibody to label pre-synaptic terminals, and DsRed and GFP antibodies to enhance the signal from the encoded expressed pre- and post-synaptic fluorophores (Figures 1B,C). Sections were then imaged and reconstructed in three dimensions (Figure 1D2). In a separate experiment we quantified the accuracy and reliability of synapse identification by AT. For this work we focused on L4 because of the higher density of TC synapses. L4 neurons were labeled with GFP using an AAV expressing FLEX-reverse GFP (Atasoy et al., 2008) injected into primary somatosensory cortex of six3-CRE mice (which express CRE recombinase in L4, but not other neocortical layers, Liao and Xu, 2008). AT fluorescence microscopy and EM were performed on the same serial sections (Figure 1D1).

### Large-scale array tomography (LSAT) of TC synapses on L5 pyramidal neurons

**Figure 2:**
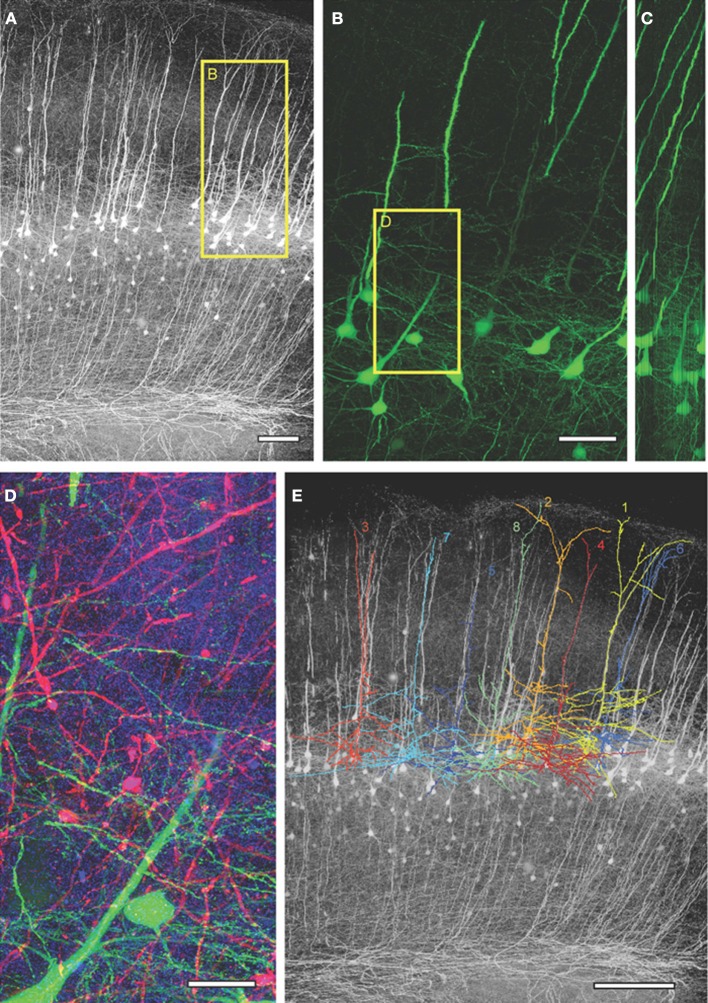
**Large-scale array tomography images from barrel cortex of Thy1-YFP_h_ mouse. (A)** Maximum intensity projected YFP image from the aligned image stack obtained from 1074 serial sections. **(B)** Maximum intensity projection of 300 serial sections (=60 μm thick) from the box in **(A)** showing YFP-expressing L5 pyramidal neurons. **(C)** Orthogonal *y*-*z* view of **(B)**. **(D)** Close up of box in **(B)**, 150 serial sections (=30 μm thick) showing VPm axons/terminals in red, YFP-expressing L5 dendrites in green and synaptophysin staining in blue. **(E)** The tracings of the eight reconstructed L5 neurons superimposed on the max projection YFP image. Scale bars: 100 μm **(A)**, 50 μm **(B)**, 20 μm **(D)**, 200 μm **(E)**.

**Figure 3:**
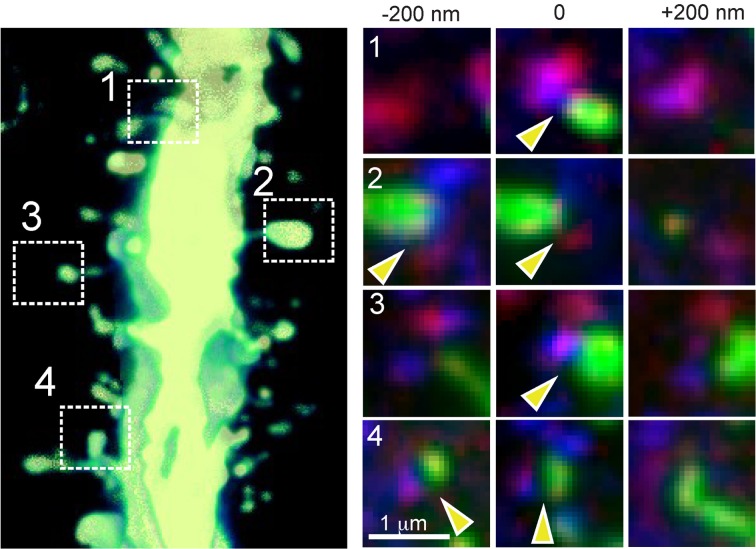
**TC synapse detection by AT.** High power view of a section of dendrite of an YFP-expressing L5 pyramidal neuron with four example thalamo-recipient spines indicated (left panel; 3-D volume rendering of 13 serial sections = 2.6 μm thick). Right panels: images from three serial sections showing spine (YFP signal, green), TC terminal (tdTomato, red) and synaptophysin staining (blue) for each of the four indicated example TC synaptic contacts. Yellow arrowheads indicate locations of identified synaptic contacts (note that for synapses 2 and 4, the synaptic contact is detected in two of the images).

**Figure 4:**
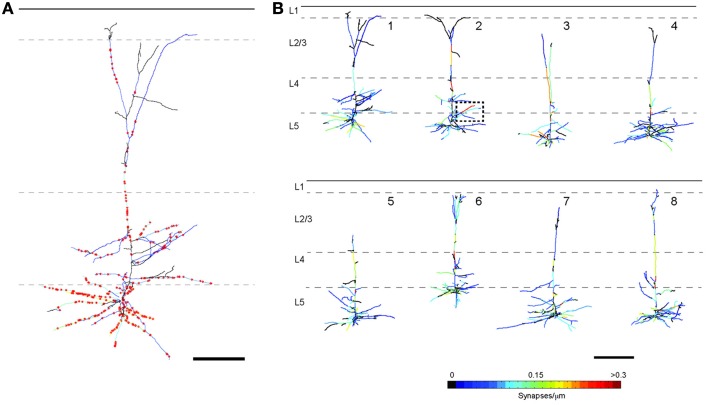
**Tracings of the eight reconstructed L5 pyramidal neurons. (A)** Enlarged image of the reconstruction of neuron 1 including positions of all TC synapses (red stars; scale bar = 100μm). **(B)** Each dendritic branch is color-coded for TC synapse density (scale bar = 200 μm).

For LSAT we prepared 1074 serial ribbon sections (thickness, 200 nm) of barrel cortex and immunostained each as above. Imaging was carried out on an automated Zeiss Observer inverted fluorescence microscope using a 100x, 1.45 N.A.objective and custom autofocussing (see Experimental procedures). Each section (area, 1.00 × 0.83 mm^2^) was imaged as a series of 240 overlapping tiles for a total of 257,760 images. The imaging time per tile was ~5.3 s and to image the entire volume required a total of ~877.5 h. The images were aligned in three dimensions using custom software and rendered in three-dimensions. The imaged volume contained the somata of ~56 labeled pyramidal neurons (Figures 2A–D; Supplementary movie 1). We chose eight neurons for manual reconstruction because large parts of their dendritic arbors were contained in the imaged volume (Figure 2E). In some neurons the apical tufts were severed. However, TC inputs from the VPm innervate L1 and L2 only sparsely (Jensen and Killackey, 1987; Zhang and Deschenes, 1998; Petreanu et al., 2009; Meyer et al., 2010; Oberlaender et al., 2012), suggesting that only a small fraction of TC input was missed. We also observed some enlargements of the TC axons, which may be result of overexpression of tdTomato. We identified putative TC synapses on these eight pyramidal neurons by manually inspecting the three-color image stacks. We scored YFP-labeled spines (green) touching tdTomato positive TC terminals (red) that were stained for synaptophysin (blue; Figure 3; see Materials and Methods). We reconstructed 4.7 ± 0.8 mm (mean ± standard deviation) dendrites and counted 328.0 ± 31.2 TC synapses per neuron (Figure 4).

### Accuracy of synapse assignment

**Figure 5:**
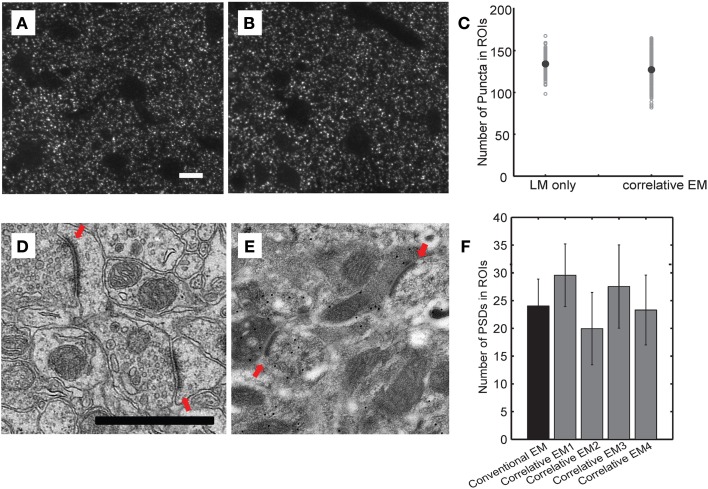
**Preparation conditions used for correlative electron microscopy preserve antigenicity while allowing reliable EM detection of synapses. (A–C)** Comparison of number of synaptophysin-positive punctae using different fixation protocols. Representative light microscopic images of synaptophysin immunostaining in sections from brains fixed using the LSAT protocol **(A)** and the correlative EM protocol **(B)**. Quantification of synaptophysin-positive punctae in the two conditions **(C)**. **(D–F)** Comparison of the number of PSDs detected in EM images under the two fixation conditions. Representative electron micrographs from sections from brains fixed using a traditional EM fixation protocol **(D)** and the correlative EM fixation protocol **(E)**. Red arrows show identified synapses. Quantifications of number of synapses detected PSD under traditional EM conditions and from four independently prepared samples using the correlative EM conditions **(F)**.

A critical unresolved question concerns the accuracy and reliability of AT in assigning synapses to the correct dendrite because of the high density of synapses in the cortical neuropil (~1/μm^3^). Although theoretical work predicts that AT can detect synapses with a high degree of accuracy (Mishchenko, 2010), this has not been rigorously tested experimentally. Thus, it is currently unknown whether AT is a truly quantitative method for synapses detection in large volume reconstruction. To address this issue, we directly compared synapse detection by AT with detection using EM on the same sections. We performed this experiment on TC inputs to L4 neurons in barrel cortex because of the higher TC synapse density in this region. Traditional EM preparation techniques quench intrinsic fluorescence of GFP and greatly reduce antigenicity. Therefore we explored the parameter space for conditions that can sufficiently preserve intrinsic fluorescence and antigenicity to allow light imaging for AT while still retaining sufficient structure in EM to reliably detect synapses. We found that a low concentration of osmium (0.001%) provided enough structural preservation for transmission EM while preserving synaptophysin antigenicity and GFP fluorescence. We compared the detection of synaptophysin punctae in this “correlative” EM condition to L4 barrel cortex sections prepared using a more traditional EM protocol (Figure 5). There was no difference in the number of synaptophysin punctae between these conditions. We also compared the number of post-synaptic densities (PSDs) detected in the same sections using EM. Although the ability to image membranes was diminished under the correlative EM conditions, PSDs were reliably detected and we consistently found no difference in the number of PSDs detected in L4 barrel cortex compared to traditional EM processing. Therefore, our correlative EM conditions allowed us to directly quantify the number of synapses detected both by AT and EM on the same ultrathin sections.

**Figure 6:**
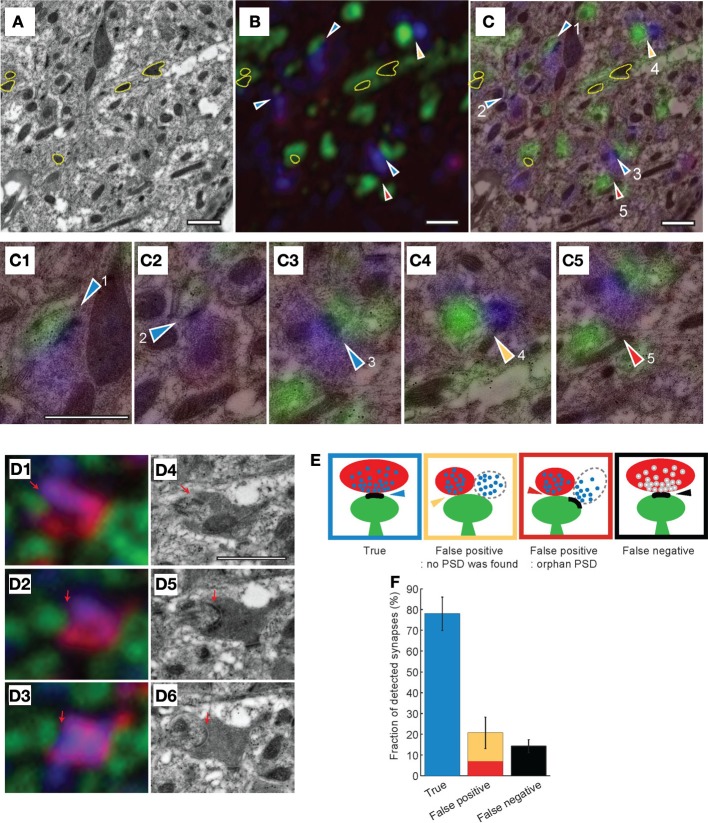
**Combined electron and light microscopy to determine the accuracy of synapse detection by array tomography. (A–C)** Transmission EM **(A)** and light microscopy **(B)** images from the same section of tissue, and the two images superimposed **(C)**. Yellow contours in all three images show mitochondria that are used as fiducial markers for aligning images. Arrowheads indicate predicted TC synapses based on the light microscopic image, colors of arrowheads show true-false evaluation based on the EM image (as depicted in **(D)**. **(C1–C5)** Close up images of TC synapses predicted by light microscopy. (**D**) Correlative light **(D1–D3)** and EM **(D4–D6)** images from the same serial sections. Red indicates thalamic axons, green postsynaptic structure, blue synaptophysin. Black dots in EM images are gold particles from immunogold staining for GFP. Red arrows indicate the PSD from the EM images used to identify. **(E)** Schematics of synapse assignments based on combined EM and light microscopy. **(F)** Quantitation of different synapse assignments based on combined EM and light microscopy (evaluation of 322 putative TC synapses). Scale bars = 1 μm.

To directly compare synapse detection by AT and EM, we acquired light and electron microscopic images from the same stacks of serial sections (Figure 6). Since mitochondria are easily identified in EM and are GFP fluorescence-negative in light microscopy, we aligned the light and EM images using the mitochondria as unambiguous and abundant landmarks (Figures 6A–D). TC synapses in the stacks of images were then manually assigned using the light microscopy images. Once completed, the EM images of the same stacks of sections were then examined (blind to the light microscopy synapse assignment) and TC synapses assigned using this imaging modality. The combined light and EM image stacks were then examined together and TC synapses were classified into three categories using the EM image as ground truth (Figures 6C,E): (1) “True,” a pre-synaptic terminal containing red (tdTomato expressed in TC axon terminal) and blue (synaptophysin staining) apposed to a green spine (post-synaptic GFP expression) exhibiting a PSD; (2) “False positive,” pre-synaptic red and blue, post-synaptic green, but no detectable PSD apposed to pre-synaptic terminal; (note that these are sub-classified into two types based on whether a PSD is detected on the spine; Figure 6E), and (3) “False negative,” a TC pre-synaptic terminal (tdTomato and synaptophysin positive detected with light microscopy) apposed to a PSD detected by EM, but this synapse is not detected in light imaging. Using this approach, we analyzed 322 putative TC synapses from 4 independent experiments and found a false positive rate of 22 ± 8.0% and a false negative rate of 14.2 ± 3.1% (Figure 6F). Therefore under our conditions, using AT alone at least 78% of TC synapses are correctly identified, and 14% are not detected.

**Figure 7:**
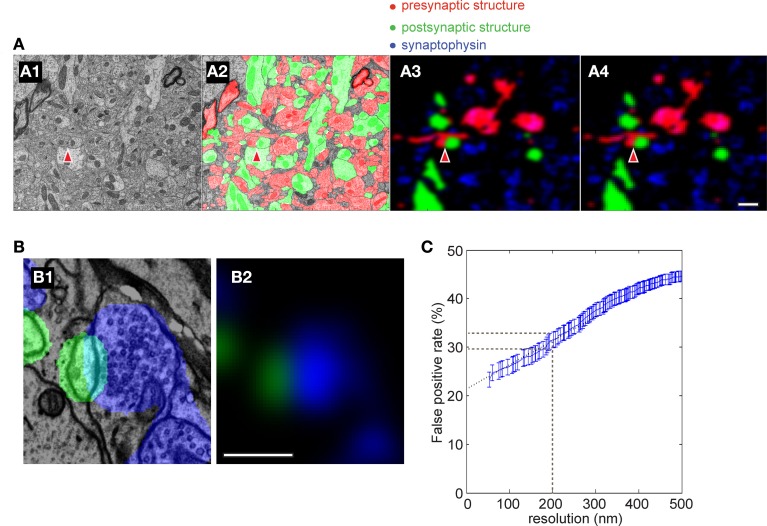
**Simulation of accuracy of synapse detection by AT. (A)** Simulated AT images using a previously segmented EM image data set [from Mishchenko et al. (2010)]. **(A1)** an example EM image; **(A2)** same image segmented into pre- and post-synaptic structures; **(A3)** segmented image was blurred to produce the same resolution as in the light microscopy of AT with 90% of structures removed to mimic the sparseness of fluorescent labeling of the AT images; **(A4)** The pixel size of A3 was adjusted to produce the same image as in the light microscopy in AT. Red indicates presynaptic structures, green postsynaptic, blue synaptophysin and red arrowheads indicates a synapse. Scale bar = 1 μm **(B)** Close-up of a simulated synapse showing postsynaptic structure (green) and simulated synaptophysin staining (blue). Note that the predicted synaptophysin staining exhibits an increasing intensity gradient toward the synaptic contact as found experimentally (Figure 10; Scale bar = 0.5 μm). **(C)** False positive rate in simulations of AT using different light imaging isotropic spatial resolutions. Black dashed line indicates resolution of the light imaging in the current study. Blue dotted line is an extrapolation of simulated data to infinite resolution.

We next compared the experimentally-determined accuracy to that obtained from a simulation of our staining and imaging conditions. We first took a stack of EM images that had been segmented (Figure 7A1) (Mishchenko et al., 2010). The segmented volume was color coded as red (pre-synaptic structure), green (post-synaptic structure) or white (synaptic contact) (Figure 7A2). To simulate the corresponding fluorescence signal that would be generated by AT over a range of simulated light imaging resolutions out of the EM image, we did the following steps. The fluorescence signal was made sparse to simulate our experimental labeling, and synaptophysin staining of pre-synaptic vesicle clusters Figures 7A3,B1; blue) was added. We then blurred the image to recreate our experimental optical resolution (Figure 7A3) and finally increased the pixel size to that of our images (Figure 7A4). We then calculated the number of synaptophysin punctae observed to be apposed to each individual spine head at the different light imaging resolutions and compared the accuracy of this detection to the EM image to calculate a false positive rate for synapse detection by light microscopy (see Materials and Methods for further details). The modeling predicted ~30% false positive rate at our imaging resolution (200 nm) (Figure 7C), similar to the ~22% false positive rate observed experimentally.

### The distribution of TC synapses on L5 pyramidal neurons

**Figure 8:**
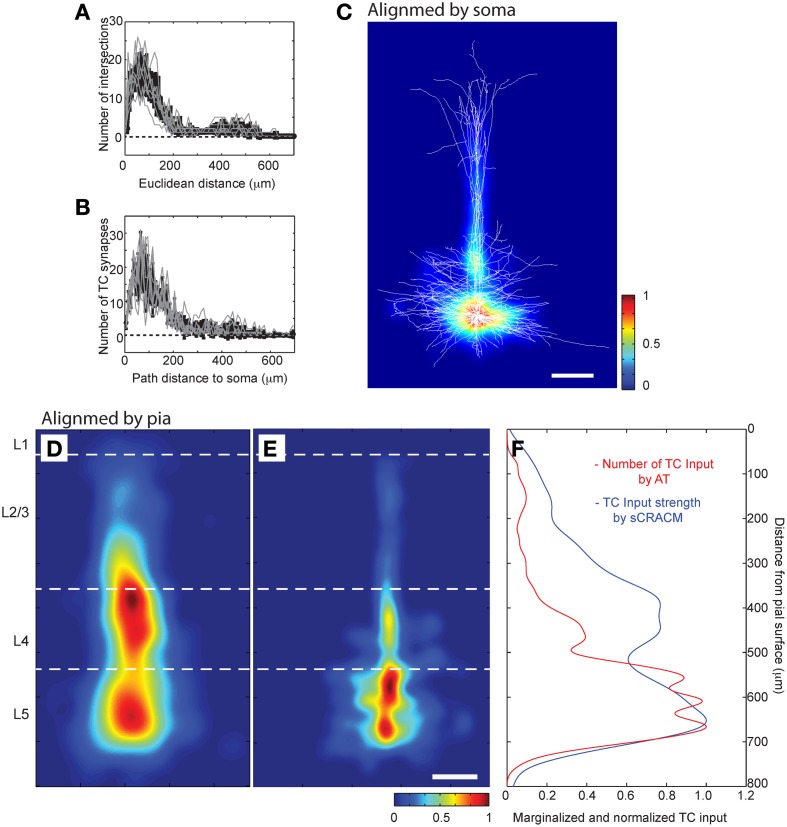
**LSAT shows that TC synapses are concentrated on proximal basal dendrites of L5 pyramidal neurons. (A)** Three dimensional Scholl analysis of dendritic structure of reconstructed L5 pyramidal neurons, and **(B)** Number of TC synapses as a function of path distance from center of soma. **(C)** Average density of TC synapses from all annotated neurons (aligned relative to cell bodies; false color scale) represented on top of all dendrites from all the traced neurons (white lines, superimposed; scale bar 100 μm). **(D,E)** Spatial distribution of the functional TC input mapped using sCRACM (Petreanu et al., 2009) **(D)**, false color scale; reduced to 80% of size to compensate for the tissue shrinkage during LSAT processing) and distribution of TC synapses detected by LSAT **(E)** average from all annotated neurons aligned relative to pia; scale bar 100 μm). **(F)** Overlay of TC laminar distribution measured by sCRACM and LSAT.

Having established the accuracy of AT in detecting synapses, we next analyzed the distribution of the TC input to L5 pyramidal neurons in our LSAT data set. We first looked at the distribution of TC synapses relative to the soma and cortical layers. Although the dendritic trees of L5 pyramidal neurons transverse L5-L1, we found that most of the TC synapses onto this cell type are located within 200 μm of the soma (Figures 8A–C; 77.9 ± 9.4% of all TC synapses within a 200 μm path length from the soma, *n* = 8). We compared our high resolution anatomical map to a functional spatial distribution map of TC inputs onto the L5 pyramidal cells in barrel cortex from our previous study using the lower resolution channelrhodopsin-2-assisted circuit mapping approach (“sCRACM”) (Petreanu et al., 2009) (Figures 8D–F). The two methods reveal a similar concentration of TC input on the basal dendrites and proximal apical dendrites. However, sCRACM shows that the TC input strength on the apical dendrites in L4 and L3 is stronger than would be predicted from the anatomy. This difference suggests that synapse strength and/or dendritic properties are additional contributors to functional TC synaptic strength in L5 pyramids. For example, there is good evidence in cortical pyramidal neurons for synaptic strength changes normalizing input along dendritic trees and active dendritic properties regulating synaptic integration that could account for the anatomical and functional differences we observe (Magee, 2000).

**Figure 9:**
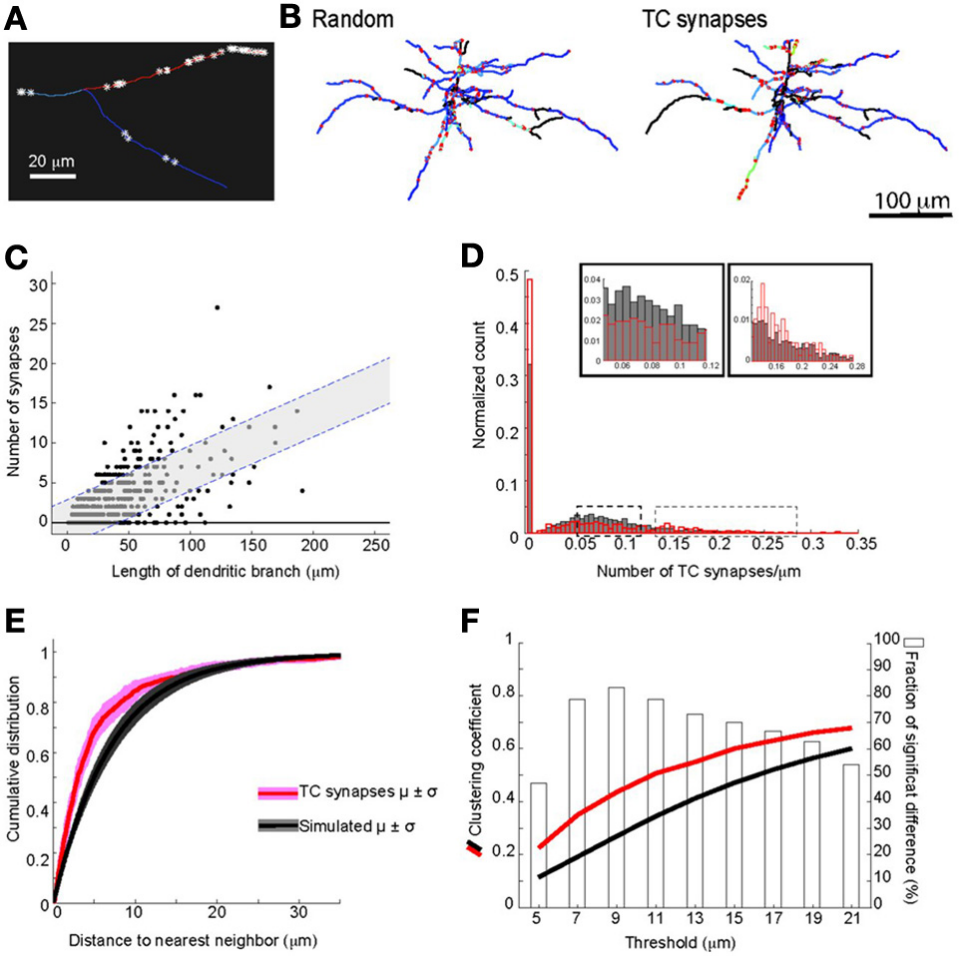
**Clustering of TC input on dendrites of L5 pyramidal neurons. (A)** Example of dendritic branches of the same L5 pyramidal cell (from dotted box in Figure 4B) showing different densities of TC synapses. **(B)** Examples of basal dendrites from simulated random synapses and the experimentally observed TC synapse distribution for the same neuron. The location of synapses is indicated by the red stars, synapse density for each branch is color-coded; view is from above (scale bar = 100 μm). **(C)** The relationships between length of dendritic branch and number of TC synapses onto L5 basal dendrites. Shaded areas with dashed lines denote 95% confidence intervals from simulated randomly distributed synapses of L5 basal dendrites. **(D)** Histogram of TC synapse density for all dendritic branches from all reconstructed neurons. Insets: expansions of peak and tail of the distribution as indicated by the dashed boxes. **(E)** Averaged cumulative distributions for nearest neighbor distances of TC synapses from L5 basal dendrites, for experimental data (red, eight reconstructed neurons) and for simulations of 8000 model neurons with randomly distributed synapses (black). **(F)** The mean clustering coefficient for TC synapses at various spatial thresholds from basal dendrites of all eight reconstructed neurons (red line) and for simulated randomly distributed synapses (black line). Bars (right y-axis) show statistical results using a KS test to determine the fraction of simulations there are significantly different from the experimental data set of whole neurons (open bars) or of L5 basal dendrites.

We next evaluated whether there was preferential targeting of certain dendritic branches by TC input. Such clustering of input on specific dendritic branches has important implications for integration of synaptic input and synaptic plasticity (Wei, 2001; Ariav et al., 2003; Polsky et al., 2004; Losonczy and Magee, 2006), but has been difficult to evaluate because of a lack of available techniques. At a coarse level it was noticeable that L5 cells do not receive TC input uniformly, with some branches receiving denser input than others (see Figure 4). To study the uniformity of TC input we compared the distribution of TC synapses on each neuron to simulated random distributions for the same neurons. One potential confound in this analysis, however, is differences in laminar distributions of TC synapses onto L5 pyramidal neurons. TC inputs from VPm onto L5 pyramidal cells occur primarily within L4, L5B, and L6 (Bernardo and Woolsey, 1987; Bureau et al., 2006; Oberlaender et al., 2012) and Figure 4. Therefore, it is possible that any apparent preference of TC synapses toward a subset of dendrites (compared to a random distribution across the whole dendritic tree) could be due to this layer-specific distribution rather than being specific to the TC input *per se*. Therefore, to control for this, we compared TC input to simulated random input onto basal dendrites only, which reside primarily within L5 (Figure 9B). If all branches have an equal probability of receiving TC input then there will be a very close relationship between dendritic branch length and TC synapse number. If not then “TC preferring” or “non-preferring” branches (e.g., blue vs. red branches in Figure 9A) will generate scatter away from the line of unity in a branch length vs. TC synapse number plot. To quantify this, we plotted the number of synapses vs. branch length from the eight reconstructed neurons (Figure 9C, closed circles) and overlaid that with the confidence interval determined from a simulated random distribution (Figure 9C, dotted lines and shaded area, 95% confidence level). 15.0% of dendritic branches were found to have a synaptic density outside the 95% confidence interval of the random distribution. This analysis shows that the experimental data set contains branches with an excess of TC synapses (above the shaded area) and a fraction with a lack of TC synapses (below shaded area) compared to the random distribution. We also evaluated preferential dendrite targeting by the TC input by plotting the normalized histogram for TC synapse density for individual dendritic branches, comparing the simulated data set for randomly distributed synapses with the experimental data set. This analysis showed that compared with the randomly distributed synapses, the experimental data exhibited a larger fraction of branches at the extremes of the distribution, i.e., that have no TC synapses or a high TC synapse density (Figure 9D, Kolmogorov-Smirnov (KS) test, significant, *p* = 1.1 × 10^−37^). These findings therefore indicate that TC afferents do not have equal preference for all basal dendritic branches on L5 pyramidal neurons but, instead, are biased toward a subset of preferred dendritic branches.

We next examined the spatial distribution of TC synapses, focusing on their distribution within individual branches of basal dendrites. We found that at this level TC synapses are not regularly distributed, but show clustering. The mean distance between neighboring TC synapses across all dendritic branches was 6.1 ± 8.7 μm, whereas a mean distance of 14.7 ± 1.9 μm would be expected if synapses are distributed regularly across all dendrites. The distribution of TC synapses within dendritic branches is different from a simulated random distribution (cumulative probability distribution of nearest neighbor distances significantly different, *p* = 9.4 × 10^−38^, *KS* statistic = 0.17; Figure 9E see Materials and Methods for detail). There is evidence for cross-talk between synapses within ~5 μm on the same dendrite that enhances the expression of synaptic plasticity (Harvey et al., 2008; Takahashi et al., 2012), and which would be particularly significant for synaptic inputs of the same type. We therefore calculated the number of TC synapses that have one or more neighboring TC synapse(s) on the same dendrite within 5 μm. We found that 67.7 ± 5.8% of TC synapses have at least one neighboring TC synapse within 5 μm, whereas a value of 50.0 ± 4.3% would be predicted for a random distribution. Thus, the clustering of TC input is predicted to enhance synaptic plasticity between TC synapses onto the same dendritic branches in L5 pyramidal neurons. To further quantify the spatial clustering, we compared the clustering coefficient of TC synapses for the experimental data set and the random distribution across a range of distances (see Materials and Methods). This analysis shows that there is a consistently greater degree of clustering for the experimental data set at distances of 5–15 μm (Figure 9F).

Together, these analyses demonstrate significant clustering of TC synapses within branches of basal dendrites of L5 pyramidal neurons, in addition to preferential targeting of certain branches by the TC input.

## Discussion

Here we characterize the use of AT for the high-resolution analysis of the distribution of synaptic input from an identified pre-synaptic source onto an identified population of post-synaptic neurons. We define the accuracy of synapse detection by AT using correlative EM imaging of the same serial sections. We go on to quantify the subcellular distribution of TC inputs onto the dendritic tree of L5 pyramidal neurons in mouse barrel cortex using LSAT. This analysis suggests that TC input targets specific dendritic branches and further that within branches it is clustered in a manner predicted to enhance plasticity and integration of TC input to L5.

### Accuracy of synapse detection by AT

**Figure 10:**
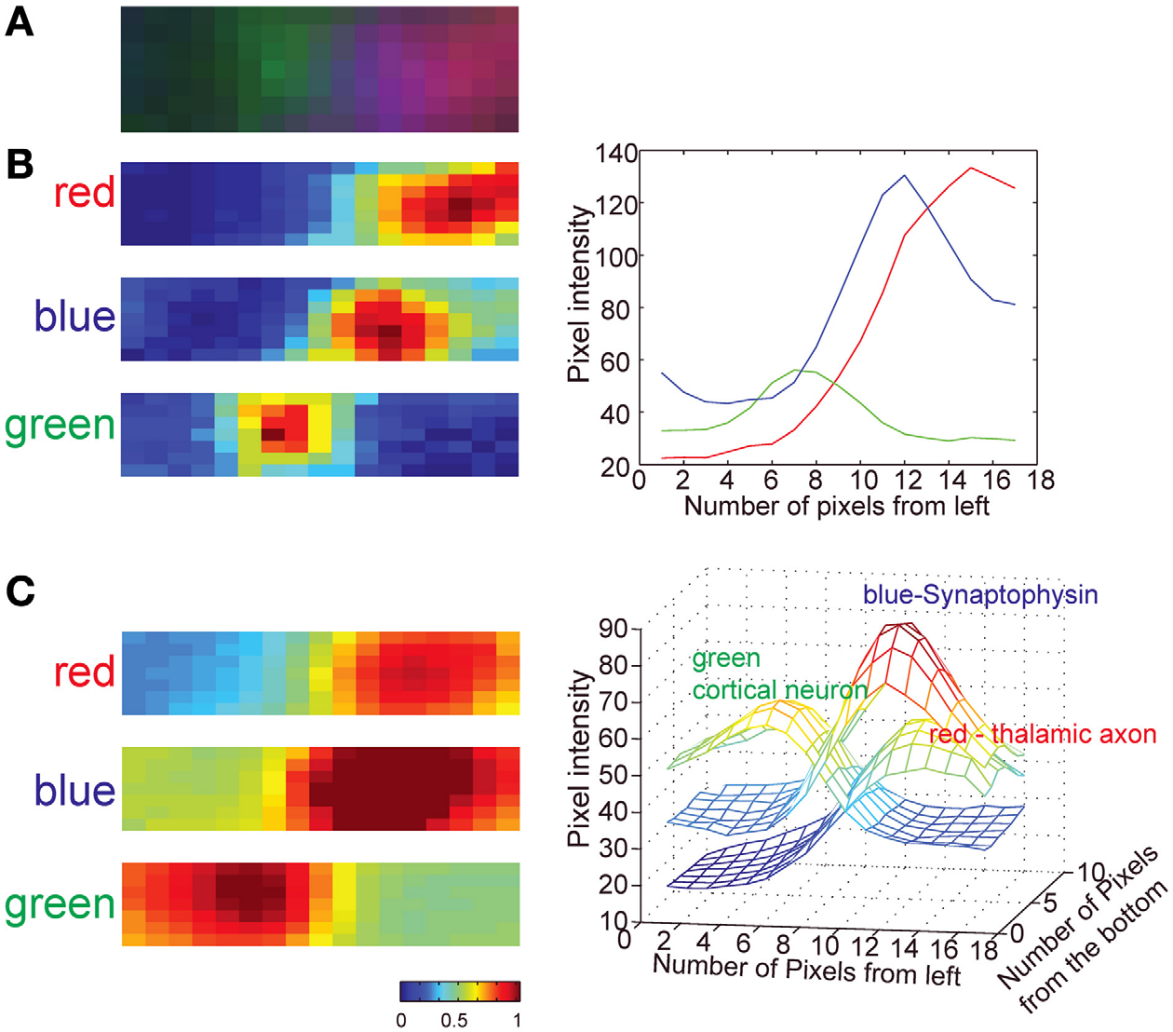
**Distribution of GFP, tdTomato and synaptophysin staining at TC synapses. (A)** Three-color image of an example TC synapse (image from a single section; green is post-synaptic GFP, red is pre-syanptic tdTomato, blue is pre-synaptic synaptophysin stain). **(B)** Pixel intensity map for each color channel for the single image shown in **(A)** (left) and the plot of pixel intensity averaged across all pixels vertically (right). **(C)** Pixel intensity map for each color channel averaged for 30 TC synapses. Individual images of synapses were first aligned based on PSDs of the correlative EM images and then cumulative signal intensity calculated. Note that the synaptophysin signal is skewed toward the presynaptic membrane whereas the tdTomato signal is not. Images shown are ~1.1 × 0.4 μm^2^.

Previous work using AT (Micheva and Smith, 2007; Micheva et al., 2010) did not quantify the accuracy or the reliability of the technique for synapse detection. A recent theoretical study (Mishchenko, 2010) suggested that an accuracy of up to 80% can be achieved using AT alone when using staining of two synaptic markers (pre- and post-synaptic) in addition to labeling of pre- and post-synaptic neurons. To address this issue experimentally, we compared for the first time the accuracy of synapse detection using AT with EM synapse detection on the same serial sections. We show, under our light imaging conditions of 200 nm isotropic resolution with three color labeling of synapses, that we achieve ~78% accuracy of synapse assignment of TC inputs to dendrites of L4 neurons. When we compared this value to the predicted accuracy of TC synapse detection using simulations, we found it to be slightly better than the predicted 70% accuracy. This difference is likely due to differences in the detection criteria used in the simulations. In the simulations we employed a simple and model-independent detection method for synapses, whereas in the experimental data synapses are detected as contacts between axons and spines, and this additional criterion has previously been shown to decrease the false-positive detection of synapses by optical image-based methods (Mishchenko et al., 2010; da Costa and Martin, 2011). Also the relatively large size of TC synapses likely further increases the accuracy of their detection (da Costa and Martin, 2011). Finally, in contrast with the assumptions in the simulation, we also observed a consistent gradient of synaptophysin staining toward the post-synaptic spine (Figure 10), reflecting an increasing concentration of synaptic vesicles with increasing proximity to the active zone. This feature provides an additional improvement in the accuracy of TC synapse detection.

We used synaptophysin immunostaining as our synaptic marker for synapse detection in AT, which labels all pre-synaptic vesicles and is one of the most abundant synaptic vesicle-specific proteins (Takamori et al., 2006; Micheva et al., 2010) (Figure 6). An alternative would have been to use labeling by a synapse associated protein (e.g., PSD-95, piccolo, bassoon) because this may increase the accuracy of synapse detection by directly labeling the pre- or post-synaptic membrane. However, immunostaining against synapse-specific proteins such as PSD-95, piccolo and bassoon has been shown to produce a relatively high false negative rate (Micheva et al., 2010). Furthermore, in our hands we were unable to quantify the accuracy and reliability of PSD-95 immunostaining with correlative EM because the antibodies were unsuitable.

In addition to quantifying false positive rates for AT, we also provide an estimate of the false negative rate for synapse detection by AT. The use of correlative EM imaging on the same sections in which light imaging was performed, allowed us to determine the number of TC synapses that we didn't detect by AT alone. These were characterized by the presence of a tdTomato- and synaptophysin-positive pre-synaptic terminal (detected by light imaging) and a PSD detected in EM, but for which no post-synaptic spine GFP signal was detected by light imaging. This evaluation provided an estimate of ~15% false negative rate. However, this is likely to be an overestimation because AT uses 3D data from serial sections and a considerable proportion of synapses are detected in 2 adjacent sections when using AT (e.g., Figure 3). Yet, in the analysis of false negatives only one section was used because of the difficulty in reconstructing the EM images in 3D due to the relatively poor membrane preservation produced using the correlative EM preparation protocol. Thus it is likely that the false negative rate for TC synapse detection is lower than 15%. Furthermore, it is likely that the undetected (false negative) synapses are small in size and therefore likely to be functionally less relevant (Matsuzaki et al., 2001; Murthy et al., 2001). The accuracy of synapse detection by AT was determined using TC synapses in L4, not in L5, because of the high abundance of TC synapses in L4. It is thus formally possible that the detection accuracy calculated using L4 TC synapses may be different to that for their detection in L5. However, we think this is unlikely because the size of dendritic spines onto L5 neurons and L4 neurons are not significantly different (Konur et al., 2003).

Thus, we find that AT can detect TC synapses with 79% accuracy and more than 85% reliability under our three-color imaging conditions. Compared to the previously reported best accuracy of 30–50% using traditional light microscopy (Sorra and Harris, 1993; da Costa and Martin, 2011), AT represents a significant improvement in synapse detection.

### The utility of LSAT for large-scale mapping of defined synaptic input

The quantification of the accuracy and reliability of TC synapse detection allowed us to map TC synapses in a large volume of reconstructed mouse barrel cortex (1.00 × 0.83 × 0.21 mm^3^). The imaging of such a large volume at such resolution presented a number of technical challenges. For example, this approach necessitated the design of a custom imaging system with customized autofocus to enable reliable imaging of the large number of sections. In addition, custom software was required for the appropriate alignment of the images. In terms of the throughput, it took 877.5 h to image the 257,760 individual image tiles from the 1074 sections in all three color channels. For comparison, to image the same volume using the current state-of-the-art EM methods, we estimate based on (Briggman and Bock, 2011) that 7656.6 h for serial section TEM using a camera array (TEMCA) (Bock et al., 2011), 21029.2 h using block-face SEM (SB-SEM) (Briggman et al., 2011) and 12455.7 h with automated tape-collecting ultramicrotome followed by SEM (Hayworth et al., 2006).

Furthermore, automated segmentation can be more readily achieved using light imaging due to the high contrast of fluorescence images. However, for AT one of the major challenges not fully resolved is the full segmentation of images to enable automated tracing of individual neurons and the automated detection of synapses. In the current study the tracing of neurons and detection and assignment of TC synapses were performed manually. More work on these aspects of image analysis will be required to facilitate a higher throughput workflow for future projects. It should be noted, however, that fully automated segmentation of images in EM reconstruction techniques also has not been achieved, and this represents one of the current biggest challenges to the practicality of any high density large-scale imaging and reconstruction technologies.

### Functional consequences of TC synapse clustering on L5 pyramidal neurons

Specialized spatial domains on dendrites preferentially targeted by certain inputs have been hypothesized to act as separately functional units for integration and plasticity (Poirazi et al., 2003a,b), providing a first layer of internal computation for neurons (Schiller et al., 2000). The consequence of such dendritic branch-level processing is that individual branches can coordinate its inputs using plasticity and can act as a computational unit representing different input features in the network (Hausser and Mel, 2003; Losonczy et al., 2008). Although there is some experimental support for this concept, it is not known whether synapses from the same pre-synaptic input can participate in local dendritic interactions to produce functional dendritic computational units representing related information from the same pre-synaptic source. We show that TC input may have appropriate anatomical features to participate in such local functionally related dendritic integration and plasticity. We show that TC inputs, carrying topographically organized sensory input preferentially target certain L5 dendrites and cluster in a manner likely to increase their functional integration and promote plasticity. Such input-specific clustering is predicted to be a powerful mechanism for circuit development promoting topographical organization of ascending sensory input.

During development, functionally clustered units of TC input would likely be promoted by synaptic plasticity in response to experience. TC axons during development are constantly edited at a high rate (35 μm/h) (Portera-Cailliau et al., 2005) to revise their synaptic connectivity in a mechanism depending on activity. Long-term synaptic plasticity during early postnatal development has been proposed as a functional maturation cue at TC synapses (Isaac et al., 1997; Feldman et al., 1999; Kidd and Isaac, 1999; Cline, 2001). Induction of long-term potentiation (LTP) at one synapse enhances the likelihood of LTP induction at neighboring synapses within ~5 μm (Govindarajan et al., 2006; Harvey and Svoboda, 2007; De Roo et al., 2008). This process provides a candidate mechanism to promote the clustering we observe for TC synapses in which there is a significant increase in the incidence of TC synapses located within 5 μm of each other (compared to a random distribution; see Figure 9D). Further support for this idea comes from findings showing that synapses are spatially organized on a fine scale to promote synchronized activity, development of which relies on NMDA receptor activation (Kleindienst et al., 2011; Makino and Malinow, 2011). In addition, a recent *in vivo* study shows that dendritic spines newly generated during learning form clusters (Fu et al., 2012). However, we cannot completely exclude the possibility that pathological enlargement of axons found in the reconstructed TC axons might have somewhat affected the distribution of TC synapses.

In addition to the local clustering, we also find that TC inputs onto L5 pyramidal neurons primarily target basal dendrites proximal to the soma (Figure 8). Together with spatial clustering, this proximal dendritic targeting likely further promotes a reliable input-output function for the TC projection, making the TC input strong and driving in L5. We found that TC inputs also target proximal apical dendrites of L5 pyramidal neurons within L4. Although these synapses are present at a somewhat more distant location from the soma than the basal dendritic targeting inputs, the large diameter proximal apical dendrites exhibit reduced attenuation of these synaptic responses making this part if the TC input also relatively strong. This is borne out by functional mapping data using sCRACM (Petreanu et al., 2009), which shows a larger functional TC response mediated by the TC input to L5 pyramidal cells onto the proximal apical dendrites in L4 than expected by the anatomic distribution we observe.

## Conclusion

In summary, we describe the use of AT for large-scale reconstruction of defined synaptic input onto defined post-synaptic neurons. We quantify the accuracy and reliability of synapse detection by AT for the first time and then show that it is suitable for the high resolution mapping the TC input onto L5 pyramidal neurons in a large volume of reconstructed barrel cortex. We find that TC synapses preferentially target certain dendrites and that TC synapses cluster in a manner predicted to enhance dendritic integration and plasticity. We anticipate that LSAT will be a highly useful tool for the quantitative mapping of connectivity in the brain, a key activity necessary for understanding information processing.

## Materials and methods

### Animals

All experimental protocols were conducted according to the United States National Institutes of Health guidelines for animal research and were approved by the Institutional Animal Care and Use Committee at the Janelia Farm Research Campus.

### Population-specific fluorescent labeling of neurons

For the LSAT experiment we used Thy1-YFP (type H) transgenic mice, in which L5 pyramidal neurons are sparsely labeled with YFP (Feng et al., 2000). In these mice (~5–6 months of age) we labeled TC synapses in the barrel field of somatosensory cortex by transducing VPm neurons with tdTomato. This was achieved by stereotaxic injection of adeno-associated virus (AAV; serotype II) expressing tdTomato under the CAG promoter and allowing ~4 weeks to achieve high levels of expression, as previously published (Petreanu et al., 2009; Hooks et al., 2011). In brief, after making a small incision in the scalp, we injected virus into the VPm of the thalamus through the thinned skull (1.45 mm posterior, 1.6 mm lateral to the Bregma and 3.1 mm deep from the pial surface). About 100 nl of viral suspension was injected through a pulled glass micropipette (Drummond, Broomall, PA). Successful targeting of VPm was confirmed by the distribution pattern of axons in S1 under low power fluorescence imaging (MVX10, Olympus, Tokyo, Japan). As previously described, neurons in L4 and L5B are the main recipients of ascending input from VPm (Bureau et al., 2006; Petreanu et al., 2009).

For the correlative light and EM study we labeled L4 neurons with GFP and TC axons and terminals using AAV-tdTomato injected in VPm. To achieve L4 specific gene delivery, we stereotaxically injected an AAV virus encoding FLEX-reversed GFP (Schnutgen et al., 2003; Atasoy et al., 2008) into L4 of a six3-CRE transgenic mouse line that expresses CRE in L4, but not other neocortical layers (Liao and Xu, 2008).

### Tissue preparation, resin embedding and ultra-thin sectioning

For correlative AT and EM, ultrathin sections had to be prepared without significant loss of fluorescence or immunoreactivity, yet with sufficient structural preservation to identify synapses unambiguously. We found the following fixation and embedding protocol meets our requirements. ~4 weeks after the stereotaxic virus injection, animals were transcardially perfused with ~200 ml of fixative (4% paraformaldehyde, 0.2% glutaraldehyde in 0.1 M sodium cacodylate buffer, pH7.2). Brains were further incubated in fixative for ~2 h before being extracted (Knott et al., 2009). Brains were then rinsed overnight with sodium cacodylate buffer and 300 μm thick sections prepared by vibratome (Leica, VT1200). The sections were then washed in cacodylate buffer and post-fixed with 0.001% osmium tetroxide in cacodylate buffer for 1 h at 4°C for correlative EM. Samples were then dehydrated by serial incubation in 30, 50, 70, and 95% ethanol series and then embedded and polymerized at −20°C in LR White using a chemical accelerator (Electron Microscopy Sciences, Hartfield, PA) in a low temperature embedding system (Leica AFS2, Leica, Buffalo Grove, IL). Embedded tissues were sectioned at 60 nm thicknesses with an ultramicrotome (Leica, Buffalo Grove, IL) and collected on Pioloform-coated Ø25-mm coverslips (Electron Microscopy Sciences, Hartfield, PA), so that the sections could be readily separated from the coverslip for subsequent EM as described in Watanabe et al. (in press). Sections mounted on Pioloform film were separated from the coverslip with diluted hydrofluoric acid aqueous solution, transferred to an EM grid and stained with 7.5% aqueous uranyl acetate followed by Sato's lead solution (Sato, 1968).

For LSAT imaging, brains were fixed by transcardial perfusion with 4% paraformaldehyde, sectioned at 300 μm, embedded in LR White and 200 nm serial sections prepared and mounted on gelatin-coated 25 × 75 mm rectangular coverslips (Electron Microscopy Sciences, Hartfield, PA) using a Histo Jumbo knife (Diatome, Switzerland).

### Immunohistochemistry

Ultrathin sections were immunostained the following antibodies: synaptophysin (1:200, Synaptic System, Göttingen, Germany), DsRed (1:500, Clontech, Mountain View, CA), GFP (1:2000, abCam, Cambridge, MA) and visualized using fluorescence-tagged secondary antibodies. Immunohistochemistry was conducted essentially as previously described (Micheva and Smith, 2007).

### Light and electron microscopy

For light microscopy we used a Zeiss Observer microscope (Zeiss, Göttingen, Germany) modified with a custom-built autofocus system. This system measures the distance between the objective and the sample surface with an auxiliary IR beam, and the sample is positioned within 100 nm of a preset target distance. Sample variation can cause changes in the distance measurement, so the edge sharpness within each image is analyzed after each acquisition. If poor edge sharpness is detected, the system acquires a *z*-stack of 15 images, and analyzes these images to determine the position of best focus for the current sample area; this newly determined target distance is used to reimage the field. A 100x, 1.45 N.A., oil-immersion objective was used for all the AT imaging. For LSAT, we collected 20 × 12 image tiles in three colors, overlapping one another by 10%, from each of 1074 sections.

For EM, samples were imaged using automated EM acquisition software, Leginon (Suloway et al., 2005) in a Tecnai Spirit transmission electron microscope (FEI, Hillsboro, OR) at 4,800x or 2,900x magnification. Images were registered at a coarse level using translational and rotational transformation in *TrakEM2* (Cardona et al., 2010) and were then aligned further using custom software. For image alignment between light microscope and EM images, images were manually aligned using mitochondria as common feature points in TrakEM2.

For LSAT, light microscopic images were aligned using custom software. The custom alignment software assigns one affine transform to each image in the stack using the following sequence of operations. All images are histogram equalized to enhance image contrast. A set of image-to-image correspondence point pairs is found for each pair of images that overlap in the same or adjacent *z*-plane. These points are found by maximizing normalized cross correlation in the overlap region using fast Fourier transforms (FFTs) and then refining the matching using a deformable mesh of triangles. The triangle centroids become the correspondence points. Next the set of all image-to-image correspondence point pairs is scanned for mutual connectivity, which determines the set of transforms {T_*i*_} sought. A large system of linear equations is constructed to express that the correspondence point pairs should map to the same place in the common global space. After solving the system using conventional matrix methods, the residual correspondence point displacements can be used to express a fit accuracy. In the current case, the R.M.S. error for the whole stack is roughly 8 pixels. The shape of the error distribution is approximately Poisson, and much of the high error tail comes from very sparse regions at the periphery of the stack where matching is poorer but precisely because content is lacking. The interesting and denser regions are sufficiently well aligned for manual tracing.

### Dendrite segmentation and TC synapse assignment

The dendritic structure of 8 neurons was manually traced using *Knossos* (Helmstaedter et al., 2011). Synapses were assigned in the AT images based on the profiles of fluorescence: TC axons were identified based on overlapping fluorescence of tdTomato and synaptophysin immunolabeling, and the post-synaptic spines were identified by YFP fluorescence. Thus, identified TC synapses in AT were selected based on the three-color channels partially overlapping. As shown in Figure 10, we used the gradient of synaptophysin signal toward the synapse as a further confirmation of the direction of synapses (Figure 10). The asymmetrical accumulation of synaptic vesicles near the active zone produces a gradient of blue synaptophysin immunostaining intensity that increases toward the active zone. In addition, because of the increasing abundance of synaptic vesicles, there is a concomitant reduction in the amount of the pre-synaptic cytosolic fluorescent label (tdTomato) with increasing proximity to the active zone. These effects combine to produce a gradient of increasing synaptophysin staining (blue) and decreasing cytosolic fluorescent protein (red) with increasing proximity to the synaptic contact enhancing TC synapse detection reliability. TC synapses were assumed to be axo-spinous (White and Rock, 1980; Benshalom and White, 1986; da Costa and Martin, 2011), therefore, thalamic afferents on large green structures, which are likely a part of dendritic shafts or soma, were not assigned as TC synapses.

The accuracy of TC synapses assignment was evaluated with the correlative EM images. The same stacks of serial sections were imaged using EM and AT, and synapses assigned independently using both imaging modalities. This analysis was performed in two experiments using stacks of 4 serial sections, in one experiment using stacks of 15 serial sections and in a further experiment using single sections. Since synaptic vesicles in pre-synaptic terminals were not visible under our correlative EM conditions, synapses in these EM images were defined by the presence of an electron dense post-synaptic density (PSD) structure. To avoid experimenter-based bias, the accuracy of synapses assignment both in light and EM was assessed independently by two scientists. Dendritic morphology was quantified using 3-dimensional Sholl analysis. The number of intersections between dendrites and concentric spheres centered at the center of the cell body were counted at various diameters using custom software.

## Simulations

For the simulations of accuracy of detection using AT (shown in Figure 7) we used serial EM images from hippocampus (Mishchenko et al., 2010). In this data, all synapses, pre-synaptic boutons, and post-synaptic spines were marked automatically and then verified manually as described in (Mishchenko, 2010). Only synapses between well defined spine heads and axonal boutons were kept for the calculation. These constituted a dataset of 250 well defined synaptic contacts. For each individual pre-synaptic bouton we constructed in *Matlab* (Mathwork, MA) the distance transform for the associated spine head using the fast anisotropic 3D distance transform Matlab function *bwdistsc* (Mishchenko, “3D Euclidean Distance Transform for Variable Data Aspect Ratio,” Matlab Central Website, 2007). Thus, the distance transform assigned the distance from each pre-synaptic bouton to each reference spine head. Using these constructed distance transforms we calculated, for each synaptic contact and each light imaging resolution, the total number of different vesicle clouds located within that resolution limit away from associated spine head. If the number of such “proximal” vesicle clouds is greater than one, that creates a possibility for incorrectly assigning a pre-synaptic axon to a post-synaptic spine thus falsely identifying a synaptic contact. A vesicle cloud was assumed to contribute to the fraction of false-positives at resolution *d* if any of its vesicles were found to be within the distance *d* away from a spine head. Using this analysis, we calculated the probability of false-positive associations for each synapse at different light imaging resolutions. Specifically, if the total number of axonal boutons proximal to a reference spine at distance *d* was *n*_*spn*(*d*), then the probability of false-positive associations in these settings was calculated using the following formula: *p*_*spn*(*d*) = (*n*_*spn*(*d*) − 1)/*n*_*spn*(*d*). The overall false-positive error rate was then evaluated as the average over all the individual synapses.

To test whether the observed distribution pattern of detected TC synapses can be generated by chance alone (data shown in Figure 9), we built model neurons that have a random distribution of synapses on the same segmented basal dendritic structure of the eight reconstructed L5 pyramidal neurons. For each of the eight neurons, we did this by randomly distributing the same number of TC synapses as measured experimentally on a line the length of the total dendritic path length of basal dendrites of the neurons. We used the random permutation function of Matlab (*randperm*), which employs uniform distribution (*rand*) to get the permutation vector; we shuffled the seed of the random generator with the CPU clock before every simulation. The line was then reconstructed back into the real dendritic structure of the neuron to produce a random distribution of TC synapses. For each neuron this simulation was performed 1000 times.

### Nearest neighbor distance and clustering coefficient

To calculate distance to the nearest neighbor for each synapse, we first established the pairwise path length constrained by the shape of neurons between all possible combinations of synapse pairs. Then for each synapses, we defined the nearest neighbor as the synapse that has shortest path length out of all synapses.

Clustering coefficient was calculated using graph theory (Bullmore and Sporns, 2009), defining two synapses as “connected,” when they are within the defined neighboring distance. The clustering coefficient of each synapse (Figure 9E) was then calculated as the ratio of the number of connected pairs between neighboring synapses to that of all possible edges within the neighborhood in basal dendrites. Clustering coefficients were averaged across all basal dendrites of eight reconstructed neurons and compared to those calculated for simulated neurons with random synapse distributions. The difference in clustering coefficient between experimental and simulated data sets was compared using a KS test.

### Statistics

To compare the experimentally observed TC synapse distribution to the random distributions, a non-parametric distribution-free Kolmogorov-Smirnov test (KS test) was used. Paired *t*-tests were used to examine whether the TC synapse distribution pattern is significantly altered by restricting the analysis on the branches within L5. *p* < 0.05 was considered as statistically significant. All errors shown in the text as well as in the figures are standard deviation.

### Conflict of interest statement

The authors declare that the research was conducted in the absence of any commercial or financial relationships that could be construed as a potential conflict of interest.

## Supplementary material

Supplementary movie 1 **Three-dimensional volume rendering of LSAT from Figure 2B**. https://docs.google.com/file/d/0Bz3vPKnEbwTZLS1URHV1M0M5Y1E/edit?usp=sharing
